# Dynamic Changes of Cytokine Profiles and Virological Markers Associated With HBsAg Loss During Peginterferon Alpha-2a Treatment in HBeAg-Positive Chronic Hepatitis B Patients

**DOI:** 10.3389/fimmu.2022.892031

**Published:** 2022-05-04

**Authors:** Minghui Li, Luxue Zhang, Si Xie, Fangfang Sun, Zhan Zeng, Wen Deng, Tingting Jiang, Xiaoyue Bi, Yanjie Lin, Liu Yang, Yao Lu, Ge Shen, Ruyu Liu, Shuling Wu, Min Chang, Leiping Hu, Jianping Dong, Wei Yi, Yao Xie

**Affiliations:** ^1^ Department of Hepatology Division 2, Beijing Ditan Hospital, Capital Medical University, Beijing, China; ^2^ Department of Hepatology Division 2, Peking University Ditan Teaching Hospital, Beijing, China; ^3^ Infectious Disease Department, Xuanwu Hospital, Capital Medical University, Beijing, China; ^4^ Division of Hepatology, Hepato-Pancreato-Biliary Center, Beijing Tsinghua Changgung Hospital, School of Clinical Medicine, Tsinghua University, Beijing, China; ^5^ Department of Infectious Diseases, Haidian Hospital, Beijing Haidian Section of Peking University Third Hospital, Beijing, China; ^6^ Department of Gynecology and Obstetrics, Beijing Ditan Hospital, Capital Medical University, Beijing, China

**Keywords:** hepatitis B surface antigen, chronic hepatitis B, functional cure, cytokine, interferon

## Abstract

**Objective:**

To explore dynamic changes of cytokines and virological markers associated with hepatitis B surface antigen (HBsAg) loss during peginterferon alpha-2a (PEG-IFN α-2a) treatment in hepatitis B e antigen (HBeAg) positive chronic hepatitis B (CHB) patients.

**Methods:**

It was a single-center prospective cohort study. HBeAg-positive CHB patients were prospectively and consecutively enrolled. Cytokines were detected at baseline, week 12 and 24 of PEG-IFN treatment. HBsAg disappearance rate was the primary evaluation index at 48 weeks of PEG-IFN treatment.

**Results:**

Among 100 patients who completed the 48-week PEG-IFN α-2a treatment, 38 patients achieved serum HBeAg disappearance, 25 patients achieved HBeAg seroconversion, 9 patients achieved functional cure, 37 patients had HBsAg decline of ≥1 log IU/ml, and 8 patients produced hepatitis B surface antibody (HBsAb). Albumin (ALB), fms-like tyrosine kinase 3 ligand (FLT3-L) and interferon-alpha2 (IFN-α2) in the clinical cure group were significantly lower than those in the non-clinical-cure group at baseline. After 12 weeks of treatment, HBsAg in the clinical cure group was significantly lower than that in the non-clinical-cure group (median 1.14 vs. 3.45 log10IU/ml, Z=-4.355, *P* < 0.001). The decrease of HBsAg and hepatitis B virus desoxyribose nucleic acid (HBV DNA) in the clinical cure group was significantly higher than that in non-clinical-cure group (median: HBsAg 1.96 vs. 0.33 log10IU/ml, Z=-4.703, *P*< 0.001; HBV DNA 4.49 vs.3.13 log_10_IU/ml, Z=-3.053, *P*=0.002). The increase of IFN-α2 in the cure group was significantly higher than that in the non-clinical-cure group (497.89 vs. 344.74, Z=-2.126, *P*=0.034). After 24 weeks of treatment, HBsAg, HBeAg, Flt3-L, and IL-10 in the clinical cure group were significantly lower than those in the non-clinical-cure group (median: HBsAg 0.70 vs. 3.15 log_10_IU/ml, Z=-4.535, P< 0.001; HBeAg 1.48 vs. 13.72 S/CO, Z = 2.512, *P* = 0.012; Flt3-l 0.00 vs 2.24 pg/ml, Z = 3.137, *P*=0.002; IL-10 0.70 vs. 2.71 pg/ml, Z=-4.067, *P* < 0.001). HBsAg decreased significantly in the clinical cure group compared with non-clinical-cure group (median 3.27 vs. 0.45, Z=-4.463, *P* < 0.001).

**Conclusion:**

Dynamic changes of cytokines and virology markers during early PEG IFN α-2a treatment were associated with HBsAg loss in HBeAg-positive CHB patients.

## Introduction

HBsAg loss, with undetectable HBeAg and HBV DNA, known as clinical cure or functional cure (1), is a goal of antiviral treatment for chronic hepatitis B (CHB) and is recommended by many guidelines (2, 3). The disappearance of HBsAg reflects a favorable state of immune control of viral infection in patients with CHB (4). Compared with CHB patients with negative HBV DNA and positive HBsAg, the disappearance of HBsAg can reduce the incidence of liver cancer by 5 times (5). Therefore, it’s essential to achieve HBsAg disappearance in patients with CHB. Although HBeAg seroconversion occurs in the state of natural infection, the spontaneous annual disappearance rate of HBsAg in CHB patients is very low (0.5% - 1.0%) due to virus inhibitory effect on the function of host immune cells (6). Regardless of nucleos(t)ide analogues (NA) treatment, disappearance rate of HBsAg is still less than 1% (7).

Interferon (IFN) therapy is the most important way to obtain the disappearance of HBsAg (8, 9). Our previous studies found that changes of virological and serological indexes could predict the disappearance of HBsAg in CHB patients treated with interferon (10, 11). If patients were given consolidation treatment of interferon for 12-24 weeks after functional cure, it was not easy to relapse (10, 12, 13). We also found that incidence of hepatitis B was positively correlated with IFN-α, and negatively correlated with transforming growth factor-beta (TGF-β) and interleukin- 10 (IL-10) (14, 15). Both TGF-β and interferon-gamma (IFN-γ) were found to be associated with efficacy of interferon (16). There are something worth investigating, such as, what are clinical and immunological characteristics of CHB patients who have functional cure after interferon treatment, and what kind of patients are more likely to be clinically cured. In this study we aimed to explore the dynamic changes of cytokine profiles and virological markers associated with HBsAg loss during peginterferon alpha-2a (PEG-IFN α-2a) treatment in HBeAg-positive CHB patients.

## Materials and Methods

### Patients and Follow-up

This was a single-center prospective cohort study. From November 2017 to November 2018, HBeAg-positive CHB patients who were willing to be treated with PEG-IFNα-2a in the Department of Hepatology Division 2 of Beijing Ditan Hospital Affiliated to Capital Medical University were prospectively and consecutively enrolled. Since November 2019, due to the impact of the Corona Virus Disease 2019 (COVID-19), no new patients have been enrolled. Virology markers, serological indicators, cytokine levels and biochemical parameters were detected at baseline and every 12 weeks during following treatment.

The criteria for enrollment were as previously (17): 1) Persistent HBsAg positivity (HBsAg ≥ 0.05 IU/ml) > 6 months; 2) HBeAg positivity (HBeAg ≥ 1.0 S/CO); 3) HBV DNA >10^4^ IU/ml; 4) ALT abnormal (≥ 80 IU/L) for more than 3 months or a history of significant liver inflammation (above G2) in examination before this study; 5) Aged from 18-65; 6) Willing to receive interferon treatment; 7) Not receiving immunosuppressants, hormones, or hepatoprotective medicines.

Exclusion criteria were: 1) Co-infection with other hepatitis virus[Hepatitis C virus (HCV), Hepatitis D virus (HDV)]; 2) Autoimmune liver diseases; 3) Other virus infections, such as Epstein-Barr virus, cytomegalovirus, *etc*; 4) Chronic alcohol abuse and/or other liver damaging drugs; 5) Mental illness; 6) Evidence of liver tumor [hepatocellular carcinoma diagnosed in clinical or alpha-fetoprotein (AFP)>100 ng/ml]; 7) Liver fibrosis and cirrhosis confirmed by Fibroscan (18); 8) Serious diseases of brain, lung, heart, kidney and other severe diseases that prevent patients from long-term follow-up; 9) Other liver diseases (metabolic disease, fatty liver, *etc*).

This study was approved by the Ethics Committee of Beijing Ditan Hospital Affiliated to Capital University of Medical Sciences (JDL-2017-034-01), and was registered with Clinical Trials (NCT03210506).

### Treatment Plan and Adjustment Strategy

After enrollment, PEG-IFNα-2a was injected subcutaneously at a dose of 180 μg weekly. After 12 weeks of treatment, patients with HBV DNA load above 10^3^ IU/ml would receive PEG-IFNα-2a in combination with entecavir (ETV) until the end of week 48. If a patient’s HBV DNA load was less than 10^3^ IU/ml at week 12, but more than 20 IU/ml at week 24, then ETV was added to PEG-IFNα-2a until week 48.

### Detection of Clinical Index, HBV DNA, HBV Serology and Plasma Cytokines

Blood routine (Sysmex Corporation, Japan), kidney function (Sekisui Medical CAL Co, LTD, Japan), liver function (Wako Pure Chemical Industries, Ltd, Japan), alpha-fetoprotein (Abbott Ireland Diagnostics Division, and Finisklin Business Park, Sligo, Ireland) were detected. HBV DNA load was detected by CobasTaqMan96 real-time quantitative PCR detection reagent (detection of off-line 20 IU/ml) (Roche, Pleasanton, CA, USA), and the detection limit of HBV DNA was <20 IU/ml. HBsAg, anti-HBs, and HBeAg were detected using Abbott Architect i2000 kits (Abbott Laboratories, Abbott Park, IL, USA). HBsAg <0.05 IU/ml was defined as HBsAg disappearance, HBsAb ≥10 mIU/L was defined as positive, HBeAg <1.0 S/CO was defined as HBeAg disappearance, and hepatitis B e antibody (HBeAb) <1.0 S/CO was defined as positive. HBsAg loss with HBeAg-negative and serum HBV DNA <20 IU/ml were defined as functional cure or clinical cure. Clinical indicators were detected at baseline and every 12 weeks during treatment.

The levels of IFN-α2, IFN-γ, IL-10, interleukin-17A (IL-17A), interleukin-6 (IL-6), FLT3-L, TGF-β and tumor necrosis factor-alpha (TNF-α) in plasma were detected with Luminex technique and analyzed by FLEXmap 3D analyzer. Cytokines were detected at baseline, week 12 and 24 of treatment.

### Statistical Analysis

All data were analyzed by SPSS 25.0 statistical software. Normal distribution data were expressed as mean ± standard deviation and independent sample *t-*test was used for comparison between two groups. Non-normal distribution data were expressed as median and quartile, while Mann-Whitney *U* nonparametric test was used. The counting data were expressed in frequency and percentage, and chi square test was used. All tests were bilateral tests with *P* < 0.05 as statistically significant. Bonferroni correction method was used to correct the test standard α when comparing the virological indexes, biochemical indexes and cytokines at 12 and 24 weeks, and the difference was statistically significant if *P* < 0.025.

We analyzed the relationship between the early (12 and 24 weeks of PEG-IFN α-2a treatment) response of virology, serology, and immunology indexes and HBsAg loss at 48 weeks of interferon treatment.

## Results

### Patients Characteristics and Outcome

**Figure 1:**
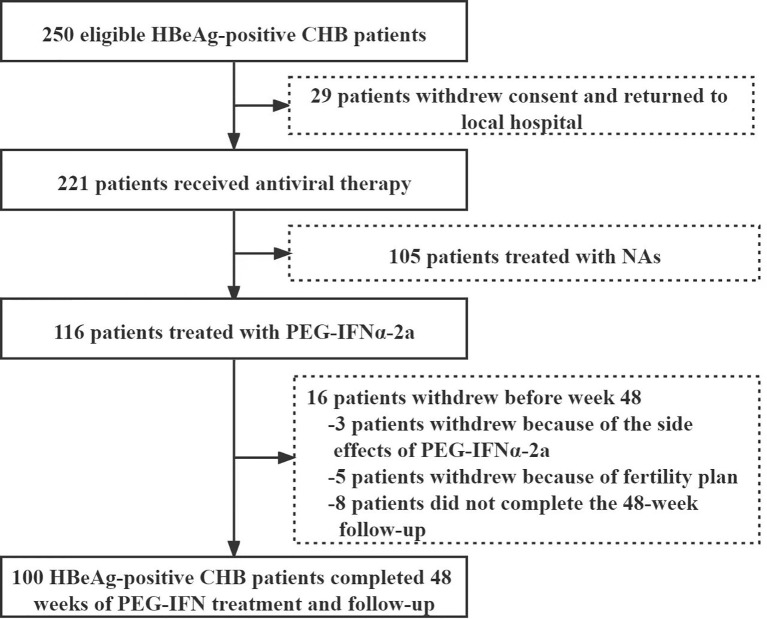
Patient enrollment and deposition.

Except 29 patients who returned to local area for treatment, 221 of 250 eligible HBeAg-positive patients with CHB infection signed the informed consent from November 2017 to November 2018. Among them, 116 were treated with PEG-IFNα-2a, while 105 were treated with NAs. 3 cases quitted from PEG-IFNα-2a group because of side effects, 5 withdrew because of fertility plan, and 8 lost to follow-up. Finally, 100 patients receiving PEG-IFNα-2a treatment completed the whole 48-week follow-up, including 60 males and 40 females, with a median age of 31 (28-36) years (**Figure 1**). 55 patients were combined with ETV therapy after peG-IFNa-2a for 12 weeks, and 10 patients were combined with ETV after PEG-IFNa-2a for 24 weeks.

After 48-weeks, 72 patients achieved virological response and 64 gained ALT normalization; 38 got serum HBeAg disappearance, and 25 achieved HBeAg seroconversion; 37 got a HBsAg decline ≥1 log10 IU/ml, 9 patients’ HBsAg disappeared, and 8 patients’ HBsAb became positive. 9 patients achieved clinical cure (HBsAg disappeared, HBeAg negative, and HBV DNA <20 IU/ml).

### Patients Grouping and Baseline Characteristics

The patients were divided into clinical cure group (*n*=9) and non-clinical-cure group (*n*=91) according to whether they obtained clinical cure (HBsAg loss, with undetectable HBeAg and HBV DNA) after 48 weeks of interferon therapy. The median levels of HBV DNA, HBeAg and HBsAg in the Clinically cure group were 6.55 log_10_IU/ml, 671.46 S/CO, 3.88 log_10_IU/ml, and those in the non-clinical-cure group were 6.69 log_10_IU/ml, 878.93 S/CO, 3.88 log_10_IU/ml, respectively, but there was no significant difference between the two groups at baseline.

At baseline, the median ALB [40.90 (39.50, 45.60) vs. 45.50 (43.10, 47.40) g/L, *Z*=-2.030, *P*=0.042], Flt3-L[0.02 (0, 28.8) vs. 29.26 (0.18, 87.92) pg/ml, *Z*=-2.080, *P*=0.037], IFN-α2 [9.13 (0.89, 52.7) vs. 43.75 (20.57, 97.83) pg/ml, *Z*=-2.187, *P*=0.029] was significantly lower in the Clinically cure group than that in the non-clinical-cure group (**Table 1**).

**Table 1:** Comparison of virology indicators, biochemistry indicators, and cytokines at baseline between Clinical cure and non-clinical cure patients.

	Clinical cure (n=9)	Non-clinical-cure (n=91)	*Z/χ^2^/P*
Age	33 (27, 43.4)	31 (28, 36)	-0.646/0.518
Male	3 (33.33%)	57 (62.60%)	1.837/0.175
HBsAg (log_10_ IU/ml)	3.88 (2.91, 4.20)	3.88 (3.61, 4.09)	-0.403/0.687
HBeAg (S/CO)	671.46 (266.45, 923.73)	878.93 (566.90, 1196.38)	-1.331/0.183
HBV DNA (log_10_ IU/ml)	6.55 (5.15, 7.29)	6.69 (6.29, 7.31)	-1.415/0.157
ALT (U/L)	271.10 (191.50, 855.01)	232.80 (123.50, 353.70)	-1.524/0.128
AST (U/L)	172.70 (63.05, 464.28)	121.50 (65.20, 168.10)	-0.753/0.452
TBil (µmol/L)	12.70 (9.80, 14.10)	14.10 (11.80, 19.00)	-1.657/0.098
ALB (g/L)	40.90 (39.50, 45.60)	45.50 (43.10, 47.40)	-2.03/0.042
Flt3-L (pg/ml)	0.02 (0, 28.8)	29.26 (0.18, 87.92)	-2.081/0.037
IFN-α2 (pg/ml)	9.13 (0.89, 52.7)	43.75 (20.57, 97.83)	-2.187/0.029
IFN-γ (pg/ml)	16.8 (8.39, 74.87)	22.75 (8.39, 63.01)	-0.416/0.678
IL-10 (pg/ml)	16.41 (1.17, 35.23)	8.9 (3.39, 18.61)	-0.163/0.871
IL-17A (pg/ml)	5.11 (2.25, 29.19)	8.64 (3.29, 39.56)	-0.596/0.551
IL-6 (pg/ml)	3.5 (0.39, 7.51)	2.03 (1.01, 8.39)	-0.271/0.786
TGF-β1 (pg/ml)	4587 (2248, 7557)	4072 (2911, 7308)	-0.633/0.527
TGF-β2 (pg/ml)	425.81 (308.41, 837.69)	499.84 (368.61, 716.21)	-0.729/0.466
TGF-β3 *Z*/*P Z*/*P*(pg/ml)	135.16 (118.63, 183.12)	155.32 (131.69, 196.55)	-0.789/0.430

### Virology, Biochemical Indices and Cytokines in Patients With or Without Clinical Cure

**Figure 2:**
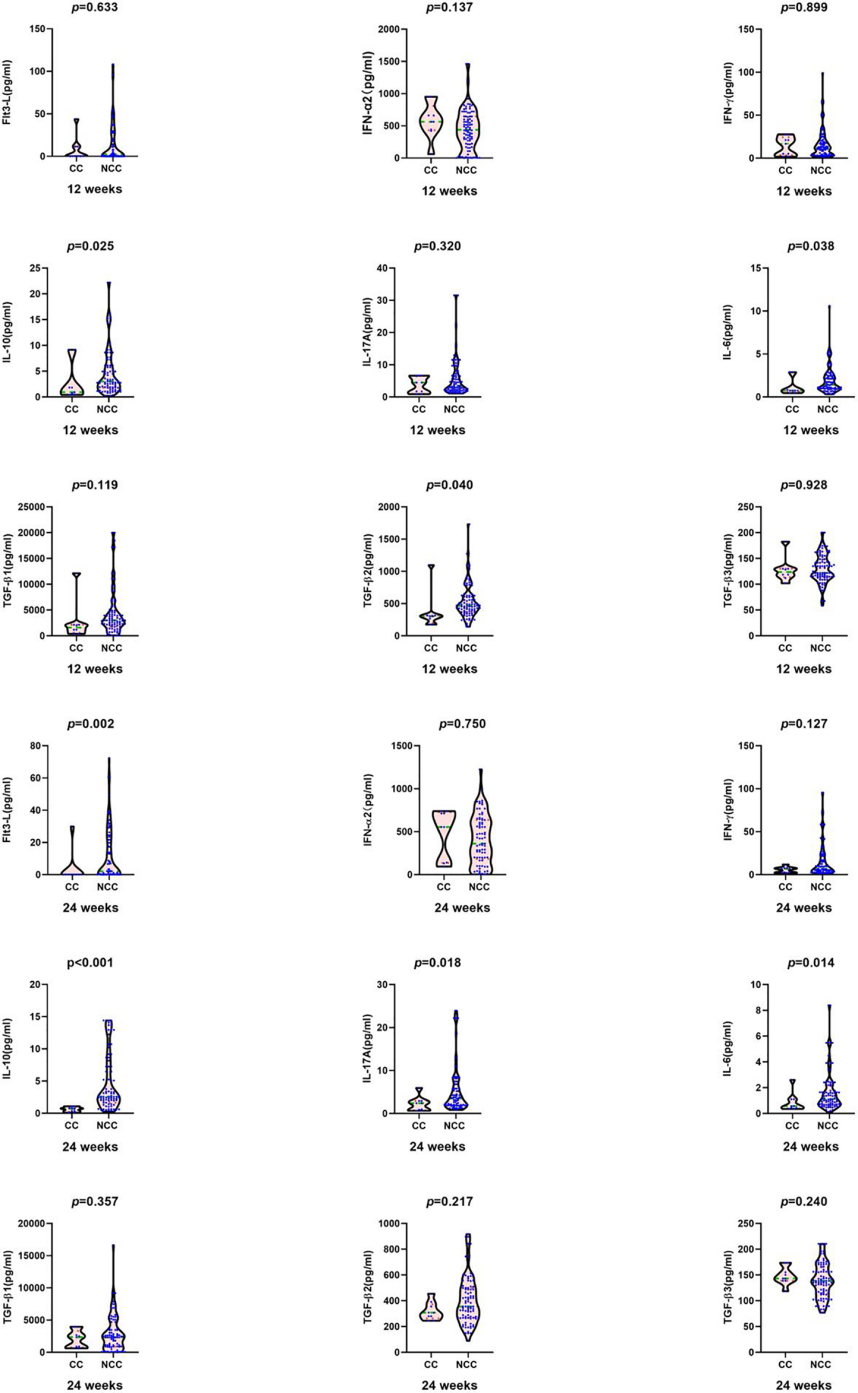
Comparison of cytokines between clinical cure group (CC: n=9) and non-clinical-cure group (NCC n=91) at week12 and 24 during interferon therapy. P < 0.025 is regarded as statistically significant. In clinical cure group, the median Flt3-L, IL-10, IL-17A and IL-6 were significantly lower than those in the non-clinical-cure group at week 24.

At 24 weeks, in clinical cure group the median HBsAg [0.70 (0.00, 1.17) vs. 3.15 (2.69, 3.74) log_10_ IU/ml, *Z*=-4.535, *P <*0.001], HBeAg [1.48 (0.32, 8.63) vs. 13.72 (2.57, 64.64) S/CO, *Z*= -2.512, *P*=0.012], Flt3-L [0.00 (0.00, 0.00) vs. 2.24 (0.04, 24.53) pg/ml, *Z*=-3.137, *P*=0.002], IL-10 [0.70 (0.22, 0.92) vs. 2.71 (1.96, 8.64) pg/ml, *Z*=-4.067, *P*<0.001], IL-17A [2.39 (0.76, 2.91) vs. 3.62 (1.9, 8.15) pg/ml, *Z*=-2.356, *P*=0.018],and IL-6 [0.57 (0.39, 1.1) vs. 1.19 (0.71, 2.29) pg/ml, *Z* = -2.464, *P* = 0.014] were significantly lower than those in the non-clinical-cure group (**Figure 2**).

**Figure 3:**
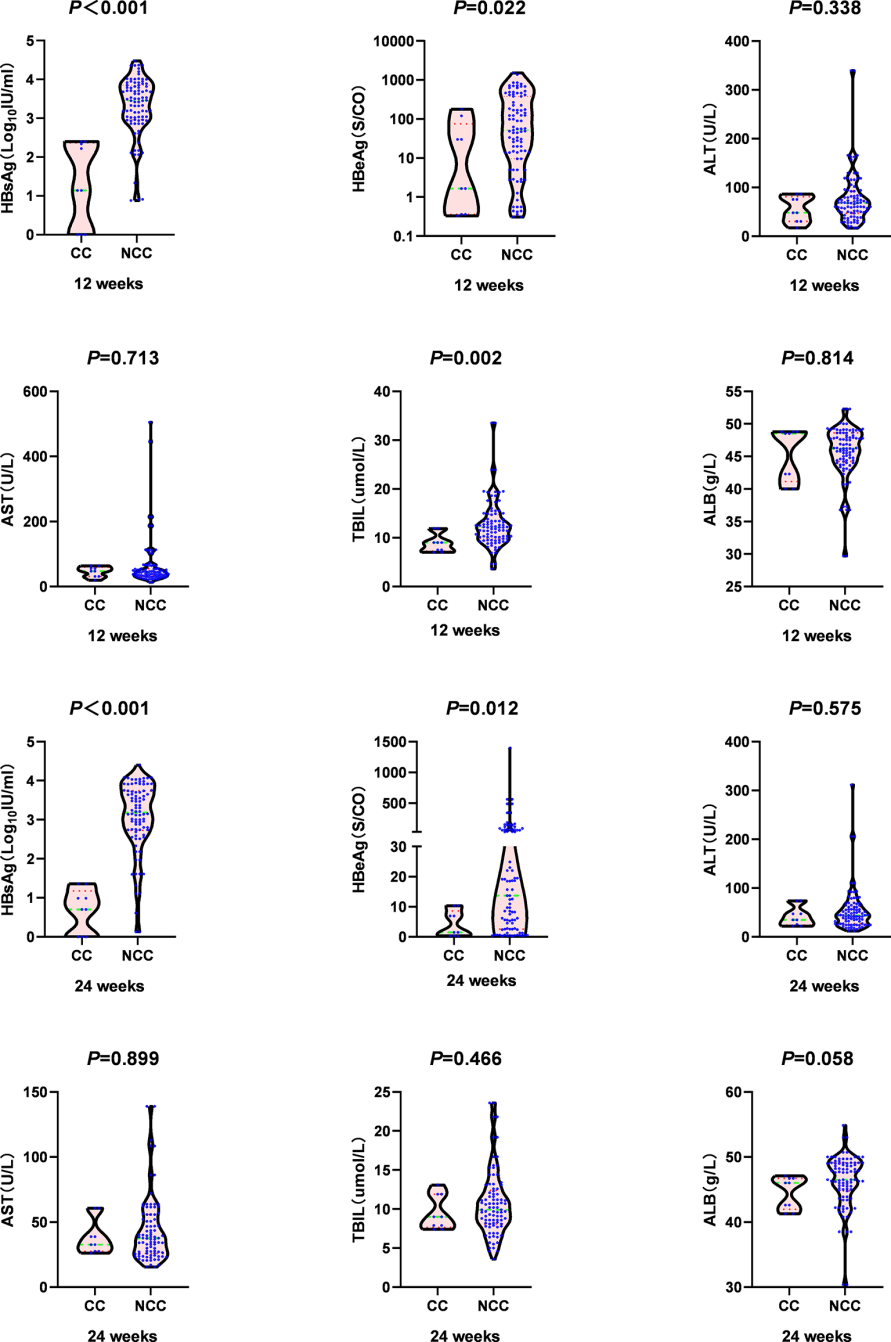
Comparison of virology indicators and biochemistry indicators at week 12 and week 24 between clinical cure (CC: n=9) and non-clinical cure (NCC: n=91) patients. In clinical cure group, the median HBsAg and HBeAg were significantly lower than those in the non-clinical-cure group at week 12 and week 24. P < 0.025 is regarded as statistically significant.

At 12 weeks, the median HBsAg [1.14 (0.00, 2.37) log_10_ IU/ml vs. 3.45 (2.96, 3.85) log_10_ IU/ml, *Z*=-4.355, *P <*0.001)], HBeAg level[1.66 (0.36, 75.20) S/CO vs.51.97 (9.60, 382.71) S/CO, Z=-2.295, *P* =0.022], and TBIL level [9.00 (7.25, 10.45) µmol/L vs. 12.10 (9.80, 14.70) µmol/L, *Z*=-3.108, *P* =0.002] in the clinical cure group were significantly lower than those in the non-clinical-cure group. The negative conversion rate of HBV DNA [5(55.60%) vs. 15(16.50%), *c^2 =^* 5.563, *P* =0.018] was significantly higher than that in the non-clinical-cure group (**Figure 3** and **Table S1**).

### Changes of Cytokines and Virological Indexes and HBsAg Response

**Figure 4:**
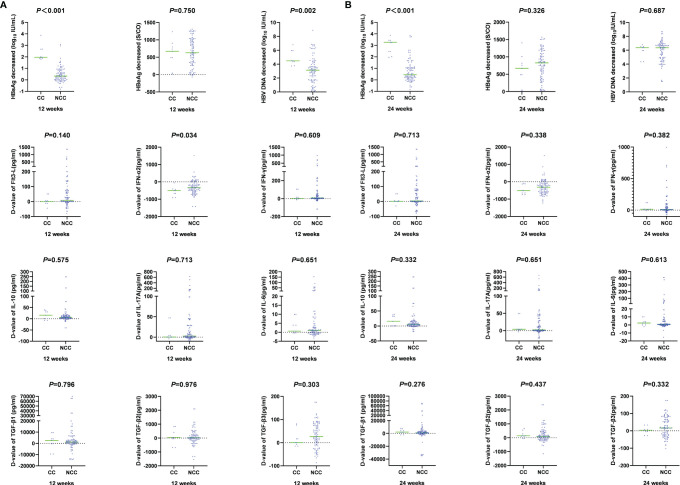
Comparison of magnitude of changes in cytokines and virological indicators at week 12 and week 24 between clinical cure (CC: n=9) and non-clinical cure (NCC: n=91) patients. A: at week 12.B: at week 24. During PEG-IFN treatment, the levels of cytokines and virological indexes changed dynamically. HBsAg, HBV DNA decreased significantly, and IFN-α2 increased significantly.

During PEG-IFN treatment, the levels of cytokines and virological indexes changed dynamically. HBsAg, HBeAg and HBV DNA decreased significantly. At 12 weeks of treatment, the decline of HBsAg and HBV DNA in the clinical cure group was significantly higher than that in the non-clinical-cure group (HBsAg: 1.96 (1.86, 2.67) vs. 0.33 (0.01, 0.81), *Z*=-4.703, *P <*0.001; HBV DNA: 4.49 (4.05, 5.95) vs. 3.13 (1.75, 4.22) log_10_ IU/ml, *Z* =-3.053, *P* =0.002). At 24 weeks, the decline of HBsAg in the clinical cure group was significantly higher than that in the non-clinical-cure group [3.27(2.24, 3.44) vs. 0.45(0.15, 1.06), Z=-4.463, *P <*0.001], as shown in **Figure 4** and **Table s2**.

**Figure 5:**
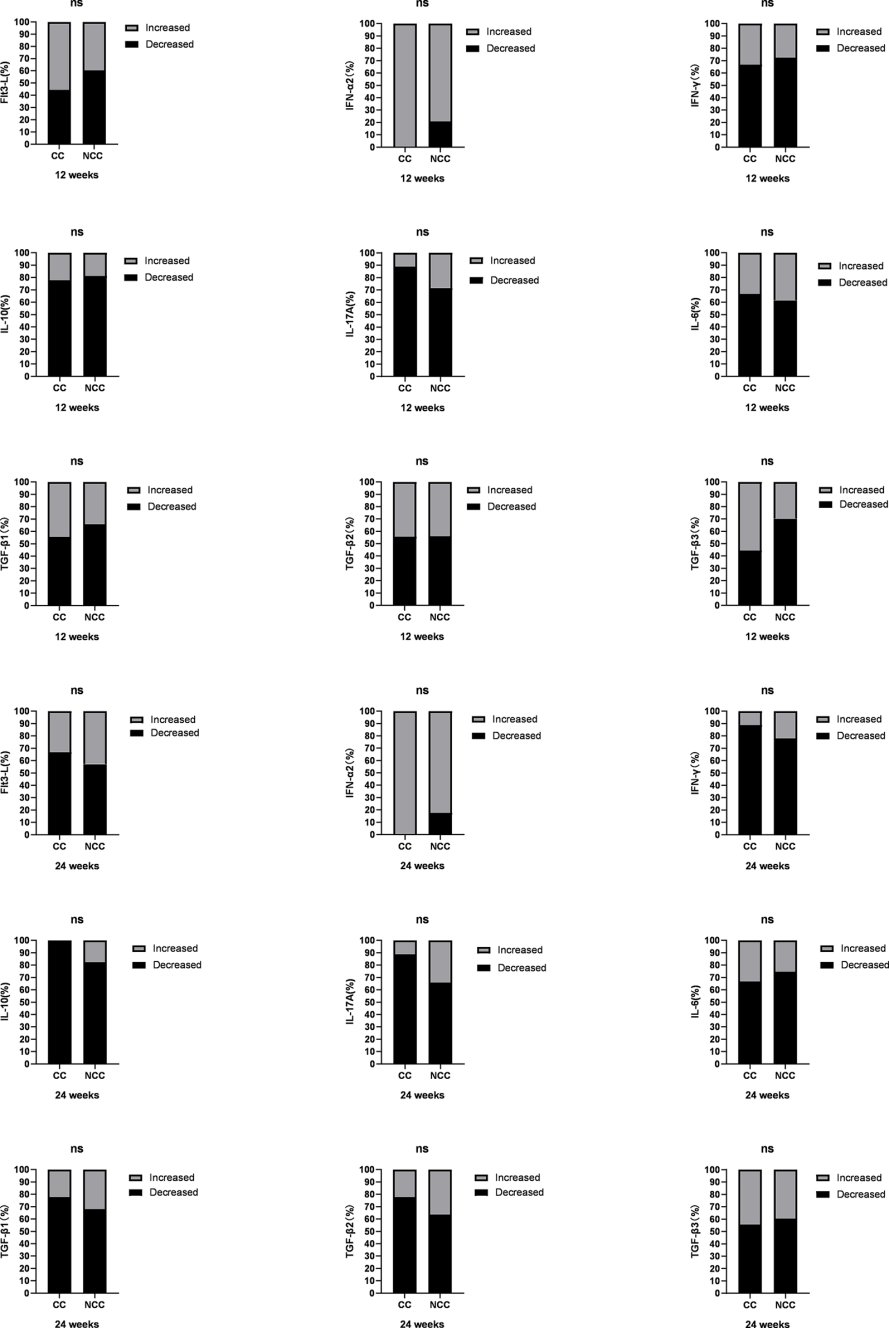
Comparison of different cytokine decline rates at week 12 and week 24 between clinical cure (CC: n=9) and non-clinical cure (n=91) patients. The decline rate of cytokines between the two groups at different times were similar. ns, no significance.

The changes of cytokines in the two groups after 12 weeks and 24 weeks of treatment were also compared. There was only significant difference in the IFN-α2 change range at 12 weeks [-497.89(-785.83, -422.21) vs. -344.74 (-542.14, -101.69), *Z*=-2.126, *P* =0.034] (**Figure 4** and **Table S2**). The decline rate of cytokines between the two groups at different times were similar (*P* > 0.05, **Figure 5** and **Table S3**).

## Discussion

With the wide application of antiviral therapy in clinic, more and more patients get HBsAg disappearance through IFN antiviral therapy (8–13). Interferon (IFN) can induce the maturation and activation of dendritic cells (DCs), stimulate the expression and cytokine secretion of natural killer cells (NK), CD4^+^ helper T lymphocytes (TH), CD8^+^ cytotoxic T cells (CTL) and monocytes, resulting in clearance of virus infected cells (16, 19). A large number of clinical data show that PEG-IFN α treatment can help 8.5%-16.2% of patients get HBsAg disappearance, especially for patients with HBsAg < 1500 IU/ml after NA treatment (11, 20–22), and the disappearance rate of HBsAg increases year by year after stopping treatment (23). Therefore, currently interferon treatment is considered as one of the most important means to get clinical cure (24). The purpose of this study was to investigate the correlation between the disappearance of HBsAg and changes of cytokine level and virological markers associated with HBsAg loss in PEG-IFN treatment.

HBsAg, HBeAg, HBV DNA polymerase and virus particles can inhibit the function of immune cells and antigen-presenting cells (25, 26), which present difficulty to obtain good curative effect through IFN antiviral therapy. Our study showed that the baseline median levels of HBV DNA, HBeAg and HBsAg in the clinical cure group were lower than those in the non-clinical-cure group. There was no significant difference between the two groups, which might be caused by the small sample size of the cure group. At baseline, the ALB level in the clinical cure group was significantly lower than that in the non-clinical-cure group. The main manifestations of liver inflammation are hepatocyte necrosis and inflammation of liver tissue. The severer the liver inflammation, the lower the ALB level. What’s more, liver inflammation leads to the reduction in HBV replication and the production site of virus antigen, i.e., clinically showing the decline of HBV DNA, HBeAg and HBsAg levels. In our study, HBsAg, HBeAg and HBV DNA decreased significantly during interferon treatment. At 12 and 24 weeks, HBsAg and HBeAg in the clinical cure group were significantly lower than those in the non-clinical-cure group, and the negative conversion rate of HBV DNA was significantly higher than that in the non-clinical-cure group. The results suggest that the more the decrease in HBV DNA load, HBeAg and HBsAg levels, the better the curative effect of interferon.

We further analyzed of HBsAg disappearance after interferon treatment by stratification to explore the correlation between immunosuppressive and immunostimulatory cytokines and the disappearance of HBsAg. Many immune cells are involved in the occurrence of CHB and interferon antiviral treatment, including CD4^+^ T lymphocytes (CD4^+^ T), CD8^+^ T lymphocytes (CD8^+^ T), myeloid dendritic cell (mDC) and plasmacytoid dendritic cell (pDC), NK, natural killer T cells (NKT) cells, monocytes, liver Kupffer cells, regulatory T cells, regulatory B cells (24). In this study we examined the cytokines with immunosuppressive effect (IL-6, IL-10 and TGF- β), cytokines with immune function stimulation (IFN -α), cytokines with virus clearance effect (IL-17A, IFN- γ), and FLT3-L stimulating proliferation of DC cells and NK cells (27–29). The expression of inhibitory cytokines (IL-6, IL-10 and TGF-β) was up-regulated, which weakened the immune response of the host. If these inhibitory cytokines are down-regulated, the host immune response will be enhanced (27–30). IFN-α is derived from pDC. IFN-α is up-regulated, and its role in the activation of NK, CD4^+^ helper cells and CD8^+^ effector cells is enhanced, and vice versa (30–32). Virus-clearing cytokines (IL-17A and IFN-γ) exert cytotoxic effects by regulating CTL cells and mediating cellular immunity. The up-regulated expression of IL-17A and IFN-γ was beneficial to elimination of the virus itself; however, if being down-regulated, it is unfavorable to the elimination of the virus (30–32). FLT3-L is an important stimulator of DC cell proliferation and can also induce NK cell proliferation *in vivo* (27, 28). Upregulation of FLT3-L enhances virus-clearance by activated immune cells, and vice versa (33).

IL-6, IL-10, and TGF-β were similar at baseline in patients with or without HBsAg disappearance, but after 24 weeks, they were significantly higher in patients without HBsAg disappearance as shown in **Figures 2**, **3** and **Table s1**. If untreated, the two groups had similar levels of inhibitory cytokines (IL-6, IL-10, and TGF-β), suggesting that the spontaneous disappearance of HBsAg is hard to occur in CHB without interferon treatment. At 24 weeks of interferon treatment, the levels of IL-10 and IL-6 in the no-HBsAg disappearance group were significantly higher than those in the HBsAg disappearance group (**Figures 2**, **3** and **Table s1**). Compared with baseline, the median decline range of IL-10 (5.21) and TGF-β2 (79.56) in no-HBsAg disappearance group was lower than that in the HBsAg disappearance group (15.71 and 145.67). As for stimulating effector cytokines (IFN-α, IL-17A, IFN-γ, and FLT3-L), the baseline FLT3-L and IFN-α2 levels in patients with HBsAg disappearance were lower than those in the no-HBsAg disappearance. At the 24th week of interferon treatment, FLT 3-L and IL-17A in the no-HBsAg disappearance were significantly higher than those in the HBsAg disappearance group (**Figures 2**, **3** and **Table s1**). Therefore, the failure of HBsAg disappearance after interferon treatment may be related to the small decline range of immunosuppressive cytokines, such as IL-10, which can inhibit the function of NK cells, PDC and HBV specific CTL cells (34).

The presence of HBsAg and HBeAg can damage immune cells, induce production of regulatory T cells, and inhibit the function of immune effector cells (14, 15, 25, 26). NA treatment cannot effectively reduce viral antigen, so it is difficult to achieve clinical cure (7). Interferon can reduce the production of viral antigens and restore the function of damaged immune cells, resulting in a higher HBsAg disappearance rate than NA treatment (12, 13, 21, 35). Our study showed that the baseline IL-10, IL-6 and TGF-β2 levels were not different in the two groups, but after 24 weeks of interferon treatment, the level of IL-10 in the clinical cure group was significantly lower than that in the non-clinical-cure group, and the clinical cure group had a greater decline range of IL-10 and TGF-β2. Our study suggests that, although interferon can stimulate immune effector cells with function of virus infected cell clearance, the clinical cure may be dependent more on the direct effect on virus replication and virus antigen reduction.

At baseline, there was a significant difference in IFN-α2 between the cured group and non-cured group, indicating that there was a difference in immune basis. But there was no significant difference between the two groups at 12 and 24 weeks of treatment owning to PEG-IFN treatment.

In conclusion, our research shows that dynamic changes of cytokine profiles and virology markers during early interferon treatment were associated with HBsAg loss in HBeAg-positive CHB patients. There was significant difference in the magnitude of changes in IFN-α2 between the two groups after 12 weeks of interferon treatment. The levels of IL-10 and IL-6 in the clinical cure group were significantly lower than those in the non-clinical-cure group after 24 weeks of interferon treatment. These characteristics help provide a basis for early screening of target population treated with interferon. Clinical cure is difficult to achieve through antiviral therapy in CHB patients. Therefore, the deviation of sample size between the cured group and the non-cured group is obviously inevitable. The bias of sample size between the two groups may affect the results of the study. It must be pointed that the study has some limitations, such as a small sample size and discrete detection range of cytokines, which needs to be further verified in future studies. For CHB patients with HBV DNA>10^4^ IU/ml, we will further explore the initial combination of PEG-IFN and NAs in the future.

## Data Availability Statement

The raw data supporting the conclusions of this article will be made available by the authors, without undue reservation.

## Ethics Statement 

The studies involving human participants were reviewed and approved by the Ethics Committee of Beijing Ditan Hospital Affiliated to Capital University of Medical Sciences (JDL-2017-034-01). The patients/participants provided their written informed consent to participate in this study.

## Author Contributions

ML, JD, WY, and YX contributed to the study design. ML, LZ, SX, and YX contributed to the data analysis. ML, LZ, SX, LY,YL, GS, RL, SW, MC, LH, and YX contributed to the recruitment, enrolment, and assessment of participants, as well as data collection. FS, WD, TJ, and XB contributed to following up with the patients. ZZ, YL, and LY managed all aspects of laboratory support. ML wrote the first draft of the manuscript. YX revised the manuscript and is the guarantor of the article. All authors contributed to the article and approved the submitted version.

## Funding

This project was supported by the Beijing Hospitals Authority Clinical medicine Development of Special Funding Support (No. XMLX 201706 and XMLX 202127), the Digestive Medical Coordinated Development Center of Beijing Hospitals Authority (No. XXZ0302 and XXT28), Special Public Health Project for Health Development in Capital (2021-1G-4061 and 2022-1-2172), Beijing Science and Technology Commission (No. D161100002716002), National Science and Technology Major Project of China (No. 2017ZX10201201-001-006 and 2017ZX10201201-002-006, and 2018ZX10715-005-003-005), and Beijing Municipal Science & Technology Commission (No. Z151100004015122).

## Conflict of Interest

The authors declare that the research was conducted in the absence of any commercial or financial relationships that could be construed as a potential conflict of interest.

The reviewer [XC] declared a shared parent affiliation with the authors [ML, LZ, FS, WD, TJ, XB, LY, YL, GS, RL, SW, MC, LH, WY, and YX] to the handling editor at the time of review.

## Publisher’s Note

All claims expressed in this article are solely those of the authors and do not necessarily represent those of their affiliated organizations, or those of the publisher, the editors and the reviewers. Any product that may be evaluated in this article, or claim that may be made by its manufacturer, is not guaranteed or endorsed by the publisher.
