# Supplementary material for: Dynamic Changes of Cytokine Profiles and Virological Markers Associated With HBsAg Loss During Peginterferon Alpha-2a Treatment in HBeAg-Positive Chronic Hepatitis B Patients
Source: Front Immunol. 2022 May 4;13:892031. doi: 10.3389/fimmu.2022.892031 (PMC9114800; doi:10.3389/fimmu.2022.892031)
Supplement: Supplementary file 1 [file Table_1.doc]

**TABLE s1.** Comparison of virology indicators and biochemistry indicators at week 12 and week 24 between Clinical cure and non-clinical cure patients

|  | **At week 12**  **Clinical cure (n=9) Non-clinical-cure (n=91) *Z*/*χ^2^*/*P*** | | | **At week 24**  **Clinical cure (n=9) Non-clinical-cure (n =91) *Z*/*χ^2^*/*P*** | | |
| --- | --- | --- | --- | --- | --- | --- |
| HBsAg (log_10_ IU/ml) | 1.14(0.00, 2.37) | 3.45 (2.96, 3.85) | -4.355/＜<0.001 | 0.70 (0.00, 1.17) | 3.15 (2.69, 3.74) | -4.535/＜<0.001 |
| HBeAg (S/CO) | 1.66 (0.36, 75.20) | 51.97 (9.60, 382.71) | -2.295/0.022 | 1.48 (0.32, 8.63) | 13.72 (2.57, 64.64) | -2.512/0.012 |
| HBV DNA (-) | 5 (55.60%) | 15 (16.50%) | 5.563/0.018 | 7 (77.80%) | 55 (60.40%) | 0.439/0.508 |
| ALT (U/L) | 48.30 (30.60, 81.15) | 68.10 (47.30, 92.10) | -0.958/0.338 | 34.90 (23.80, 60.70) | 43.90 (26.90, 60.80) | -0.56/0.575 |
| AST (U/L) | 47.00 (31.00, 59.60) | 43.70 (31.60, 55.10) | -0.367/0.713 | 32.70 (27.60, 49.75) | 37.40 (25.60, 55.80) | -0.126/0.899 |
| TBil (µmol/L) | 9.00 (7.25, 10.45) | 12.10 (9.80, 14.70) | -3.108/0.002 | 9.00 (7.65, 11.90) | 9.90 (8.10, 12.30) | -0.729/0.466 |
| ALB (g/L) | 48.50 (41.15, 48.80) | 46.20 (44.20, 48.60) | -0.235/0.814 | 46.00 (41.95, 46.70) | 46.50 (44.80, 49.10) | -1.898/0.058 |
| Flt3-L (pg/ml) | 0.21 (0.08, 11.56) | 2.34 (0, 27.82) | -0.478/0.633 | 0.00 (0.00, 0.00) | 2.24 (0.04, 24.53) | -3.137/0.002 |
| IFN-α2 (pg/ml) | 561.69 (431.33, 806.95) | 442.33 (224.32, 645.01) | -1.488/0.137 | 554.67 (137.29, 726.98) | 471.03 (197.82, 704.36) | -0.319/0.750 |
| IFN-γ (pg/ml) | 16.83 (3.50, 24.46) | 11.90 (3.62, 20.57) | -0.126/0.899 | 6.85 (2.21, 7.69) | 7.12 (3.62, 22.75) | -1.524/0.127 |
| IL-10 (pg/ml) | 0.92 (0.48, 5.51) | 3.03 (1.80, 7.26) | -2.235/0.025 | 0.70 (0.22, 0.92) | 2.71 (1.96, 8.64) | -4.067/＜<0.001 |
| IL-17A (pg/ml) | 4.48 (1.33, 6.57) | 3.50 (2.12, 8.15) | -0.994/0.320 | 2.39 (0.76, 2.91) | 3.62 (1.90, 8.15) | -2.356/0.018 |
| IL-6 (pg/ml) | 0.74 (0.61, 1.87) | 1.53 (0.98, 2.59) | -2.078/0.038 | 0.57 (0.39, 1.10) | 1.19 (0.71, 2.29) | -2.464/0.014 |
| TGF-β1 (pg/ml) | 2014.00 (791.92, 7153.5) | 3007.00 (1867.00, 4895.00) | -1.56/0.119 | 2366.00 (765.67, 3327.00) | 2556.00 (930.27, 5514.00) | -0.922/0.357 |
| TGF-β2 (pg/ml) | 303.55 (224.83, 706.65) | 467.82 (368.61, 623.44) | -2.054/0.040 | 307.55 (262.68, 373.13) | 355.05 (268.62, 499.84) | -1.235/0.217 |
| TGF-β3 (pg/ml) | 128.51 (115.35, 156.37) | 121.91 (114.56, 148.56) | -0.09/0.928 | 143.18 (138.80, 164.62) | 138.49 (114.73, 165.54) | -1.175/0.240 |

**Note:** *P* < 0.025 is regarded as statistically significant.
