# Supplementary material for: Dynamic Changes of Cytokine Profiles and Virological Markers Associated With HBsAg Loss During Peginterferon Alpha-2a Treatment in HBeAg-Positive Chronic Hepatitis B Patients
Source: Front Immunol. 2022 May 4;13:892031. doi: 10.3389/fimmu.2022.892031 (PMC9114800; doi:10.3389/fimmu.2022.892031)
Supplement: Supplementary file 2 [file Table_2.doc]

| **TABLE s2.** Comparison of magnitude of changes in cytokines and virological indicators at week 12 and week 24 between clinical cure and non-clinical cure patients | | | | | | |
| --- | --- | --- | --- | --- | --- | --- |
|  | **Magnitude of changes at week 12**  Clinical cure(n=9) Non-clinical-cure (n=91) *Z*/*P* | | | **Magnitude of changes at week 24**  Clinical cure(n=9) Non-clinical-cure (n=91) *Z*/*P* | | |
| HBsAg decreased (log_10_ IU/ml) | 1.96(1.86, 2.67) | 0.33(0.01, 0.81) | -4.703/<0.001 | 3.27(2.24, 3.44) | 0.45(0.15, 1.06) | -4.463/<0.001 |
| HBeAg decreased (S/CO) | 671.13(265.44, 848.54) | 629.9(334.52, 982.68) | -0.319/0.750 | 671.13(265.56, 915.11) | 821(429.24, 1094.62) | -0.982/0.326 |
| HBV DNA decreased (log_10_ IU/ml) | 4.49(4.05, 5.95) | 3.13(1.75, 4.22) | -3.053/0.002 | 6.37(5.15, 6.55) | 6.35(4.75, 6.82) | -0.404/0.687 |
| D-value of Flt3-L | -0.19(-11.56, 28.72) | 7.86(-2.51, 72.2) | -1.476/0.140 | 0.02(0, 28.8) | 3.17(-2.24, 75.38) | -0.367/0.713 |
| D-value of IFN-α2 | -497.89(-785.83, -422.21) | -344.74(-542.14, -101.69) | -2.126/0.034 | -513.07(-694.76, -128.17) | -314.87(-617.69, -96.91) | -0.958/0.338 |
| D-value of IFN-γ | 3.19(-3.89, 59.85) | 6.63(-0.65, 51.17) | -0.512/0.609 | 14.1(6.57, 67.6) | 8.65(1.44, 60.82) | -0.873/0.382 |
| D-value of IL-10 | 16(-3.66, 33.93) | 5.87(1.02, 13.98) | -0.56/0.575 | 15.71(0.61, 34.65) | 5.21(0.43, 14.22) | -0.97/0.332 |
| D-value of IL-17A | 0.31(0.19, 25.63) | 2.68(-0.29, 28.14) | -0.367/0.713 | 3.94(1.44, 26.64) | 2.28(-0.39, 29.05) | -0.452/0.651 |
| D-value of IL-6 | 0.61(-0.39, 6.9) | 1.05(-0.52, 4.71) | -0.452/0.651 | 2.4(0, 7) | 0.69(-0.1, 4.79) | -0.506/0.613 |
| D-value of TGF-β1 | 2573(-4905.5, 6765.08) | 1097(-516, 3287) | -0.259/0.796 | 2086(-198.45, 5914.28) | 913(-563, 3310) | -1.09/0.276 |
| D-value of TGF-β2 | 45.39(-359.81, 615.36) | 23.97(-155.23, 238.2) | -0.03/0.976 | 145.67(32.03, 414.56) | 79.56(-83.18, 395.17) | -0.777/0.437 |
| D-value of TGF-β3 | 0(-10.64, 46.5) | 26.65(-6.52, 74.64) | -1.03/0.303 | 3.29(-15.74, 21) | 16.52(-21.26, 67.21) | -0.97/0.332 |
|  |  |  |  |  |  |  |

Note: *The values at baseline minus the values of different time is D-value.
