# Supplementary material for: Dynamic Changes of Cytokine Profiles and Virological Markers Associated With HBsAg Loss During Peginterferon Alpha-2a Treatment in HBeAg-Positive Chronic Hepatitis B Patients
Source: Front Immunol. 2022 May 4;13:892031. doi: 10.3389/fimmu.2022.892031 (PMC9114800; doi:10.3389/fimmu.2022.892031)
Supplement: Supplementary file 3 [file Table_3.doc]

| **TABLE s3.** Comparison of different cytokine decline rates at week 12 and week 24 between clinical cure and non-clinical cure patients | | | | | | |
| --- | --- | --- | --- | --- | --- | --- |
|  | **Decline rates at week 12**  Clinical cure (n=9) Non-clinical-cure (n=91) *χ^2^*/*P* | | | **Decline rates at week 24**  Clinical cure(n=9) Non-clinical-cure (n=91) *χ^2^*/*P* | | |
| Flt3-L decreased, n(%) | 4(44.40%) | 55(60.40%) | 0.331/0.565 | 6(66.70%) | 52(57.10%) | 0.039/0.843 |
| IFN-α2 decreased, n(%) | 0(0.00%) | 19(20.90%) | 1.162/0.281 | 0(0.00%) | 16(17.60%) | 0.803/0.37 |
| IFN-γ decreased, n(%) | 6(66.70%) | 66(72.50%) | 0/＞0.999 | 8(88.90%) | 71(78.00%) | 0.112/0.738 |
| IL-10 decreased, n(%) | 7(77.80%) | 74(81.30%) | 0/＞0.999 | 9(100.00%) | 75(82.40%) | 0.803/0.37 |
| IL-17A decreased, n(%) | 8(88.90%) | 65(71.40%) | 0.536/0.464 | 8(88.90%) | 60(65.90%) | 1.069/0.301 |
| IL-6 decreased, n(%) | 6(66.70%) | 56(61.50%) | 0/＞0.999 | 6(66.70%) | 68(74.70%) | 0.016/0.899 |
| TGF-β1 decreased, n(%) | 5(55.60%) | 60(65.90%) | 0.066/0.798 | 7(77.80%) | 62(68.10%) | 0.048/0.827 |
| TGF-β2 decreased, n(%) | 5(55.60%) | 51(56.00%) | 0/＞0.999 | 7(77.80%) | 58(63.70%) | 0.227/0.634 |
| TGF-β3 decreased, n(%) | 4(44.40%) | 64(70.30%) | 1.473/0.225 | 5(55.60%) | 55(60.40%) | 0/＞0.999 |
|  |  |  |  |  |  |  |
